# *Rhachotropis* (Eusiroidea, Amphipoda) from the North East Atlantic

**DOI:** 10.3897/zookeys.731.19814

**Published:** 2018-01-23

**Authors:** Anne-Nina Lörz, Anne Helene S. Tandberg, Endre Willassen, Amy Driskell

**Affiliations:** 1 University of Hamburg, CeNak, Centre of Natural History, Zoological Museum, Martin-Luther-King-Platz 3, 20146 Hamburg, Germany; 2 University of Bergen, University Museum, Department of Natural History, PO Box 7800, 5020 Bergen, Norway; 3 National Museum of Natural History, Smithsonian Institution, Washington, DC, USA

**Keywords:** Amphipoda, Eusiridae, North Atlantic, IceAGE, NorAmph, COI, 16S

## Abstract

The genus *Rhachotropis* has the widest geographic and bathymetric distribution of all amphipod genera worldwide. Molecular and morphological investigations of specimens sampled around Iceland and off the Norwegian coast allow the first insights into the relationships of North East Atlantic *Rhachotropis*. The 31 cytochrome oxidase subunit I (COI) sequences generated for this study were assigned 13 Barcode Index Numbers (BINs) in the Barcode of Life database (BOLD), of which 12 are new to the database. Molecular analyses of COI and 16S sequences could not confirm a theory that depth has a greater influence on the phylogeny of *Rhachotropis* than geographic distance. Although the North East Atlantic is a well-studied area, our molecular investigations revealed the genus *Rhachotropis* may contain cryptic species, which indicates a higher biodiversity than currently known. For example, the specimens which key to *Rhachotropis helleri* is a complex of three COI clades, two of which cannot be identified with morphological traits. One specimen of each of the clades in the cladogram was documented by high definition photographs. A special focus was on the visual morphology of the eyes, as this character shows interspecific differences within the genus *Rhachotropis* in response to fixation in ethanol. Detailed morphological investigation showed that some clades thought to be indistinguishable can be separated by minute but consistent morphological characters. Datamining Genbank to examine all registered COI-sequences of *R. aculeata*, the only previously known *Rhachotropis* BIN in the North Atlantic and sub-Arctic, showed *R. aculeata* to be subdivided by an Arctic and a North Atlantic population.

## Introduction

Eusiridae are fast moving predators with a worldwide distribution. The genus *Rhachotropis* has the widest geographic (all oceans) and bathymetric (0–9460 m) distribution of all amphipod genera (Lörz et al. 2012). Currently, the genus *Rhachotropis* (Eusiridae) contains 61 species (World Amphipod Database, Horton et al. 2017).

Expeditions to the North East (NE) Atlantic via the programs IceAGE (Icelandic Animals Genetics & Ecology), Mareano and several smaller Norwegian mapping projects (Norwegian and Spitsbergen economic waters) sampled various Amphipoda during the last 10 years. Almost all amphipod collections yielded members of Eusiridae. *Rhachotropis* was the dominant genus in eusirid samples, along with three other genera: *Eusirus*, *Cleonardo* and *Eusirella*.

As the material was suitable for DNA analysis we investigated the relationships of freshly collected *Rhachotropis* from the NE Atlantic to each other via analysis of the cytochrome oxidase I (COI) and 16S gene regions. We then set these new specimens in context to *Rhachotropis* from Norway and other parts of the world.

Twenty-one of the 61 described *Rhachotropis* species are known from the NE Atlantic and Arctic region: *Rhachotropis aculeata* (Lepechin, 1780); *R. arii* Thurston, 1980; *R. distincta* (Holmes, 1908); *R. faeroensis* Stephensen, 1944; *R. gislii* Thurston, 1980; *R. gloriosae* Ledoyer, 1982; *R. gracilis* Bonnier, 1896; *R. grimaldii* (Chevreux, 1887); *R. helleri* (Boeck, 1971); *R. inflata* (Sars, 1883); *R. aff. kergueleni* Stebbing, 1888; *R. leucophthalma* Sars, 1883; *R. lomonosovi* Gurjanova, 1934; *R. macropus* Sars, 1883; *R. northriana* d’Udekem d’Acoz, Vader & Legezynska, 2007; *R. oculata* (Hansen, 1887); *R. palporum* Stebbing, 1908; *R. proxima* Chevreux, 1911; *R. rostrata* Bonnier, 1896; *R. thordisae* Thurston, 1980; *R. thorkelli* Thurston, 1980.

Lörz et al. (2012) hypothesized that depth might have a greater influence on the phylogeny of the genus *Rhachotropis* than geographic distances. The large amount of freshly collected *Rhachotropis* from the NE Atlantic allowed us to test this theory. Here we consider this theory using new data from specimens from 110 m to 2750 meters depth in the NE Atlantic. We ask and discuss the following questions:

1) What are the phylogenetic relationships of NE Atlantic and Arctic *Rhachotropis*?

2) Do relationships among specimens from the shallow (Norwegian Channel) and deep (Icelandic Basin and Norwegian Sea) stations indicate biogeographic processes such as submergence or emergence?

3) Does depth have a bigger influence on the phylogeny of *Rhachotropis* than geographic distance?

## Material and methods

The detailed description of the Icelandic study area is presented in the introduction of this volume (Brix et al. 2018). Large amphipod specimens were immediately sorted on deck, fixed in 98% ethanol and later transferred to 96% ethanol. Samples were decanted and the decant bulk fixed. The Norwegian amphipods were collected as parts of several mapping and teaching cruises and all examined through the Norwegian Biodiversity Information Centre financed project NorAmph at the University of Bergen. Material from the mapping projects “Mareano” and “Sognefjord” were sampled using RP-sleds where the material was decanted immediately and the decant bulk-fixed in 97% ethanol (Buhl-Mortensen et al. 2015). The high-arctic material from the Spitsbergen region was collected during student-cruises at the University Centre of Svalbard. Samples were sorted by teachers and students before being stored in 96% ethanol and kept in the cold-room for transport to land.

### Morphological identification and documentation

Specimens were examined and dissected under a Leica MZ12.5 stereomicroscope. Small appendages (e.g. mouthparts, uropods, telson) were temporarily mounted in glycerin and examined using a LeicaDM2500 compound microscope. The body lengths of specimens examined were measured by tracing an individual’s mid-trunk lengths (tip of the rostrum to end of telson).

Photos of material held at the Deutsches Zentrum für Marine Biodiversität (DZMB) were taken with a Canon EOS 5 Mark III with a Canon MP-E65 macro lens mounted for stacking. The stacking programme software used was Zerene Stacker 1.04 (setting P-max). Photos of the Norwegian material (all stored at the University Museum of Bergen) were assembled using a Leica DFC425 camera fitted with a motorised stacker on a Leica M205 binocular, and Leica LAS 3.8 software for taking photos. Compilation of stacked photos was done with Zerene Stacker 1.04 (P-max). Larger specimens were photographed using a Canon EOS 60D with Canon MP-E-65 (f2.8) lens.

In order to examine the eye, *Rhachotropis oculata* AMPIV228-17 was selected for confocal laser scanning microscopy (CLSM). To produce auto-fluorescence of the surfaces, 405 nm laser lines with emission filters set to 421–499 nm and 488 nm laser lines with filters set to 489 –607 nm were used. The head was scanned using a Leica DM2500 with a Leica TCS SPE at a resolution of 2480 × 2480 pixels at 10×. The software package LEICA LAS X was used for recording the image from the scans, the topmost seven photo-stack layers were removed to make the ommatidia visible. The image stacks were further processed and finalized in Adobe Photoshop CS5.

IceAGE material is held at the Zoological Museum University of Hamburg, Centre of Natural History (CeNak), Germany.

NorAmph material is held at the University Museum of Bergen, Natural History Collections, Norway.

### DNA extraction and analyses

DNA was extracted from the IceAGE specimens using an Autogen Prep 965 phenol-chloroform automated extractor following the manufacturer’s protocol for animal tissue. The barcode region of COI gene was amplified using primer pair jgLCO1490/jgHCO2198 (Geller et al. 2013) or the pair COIFamp (TTTTCTACTAAYCAYAAAGATATYGG) and COIRamp (TAAACTTCIGGGTGICCAAAAAAYC) (K. Macdonald, pers. comm.). The PCR reaction mix was comprised of 0.3 µM of each primer, 0.1 uL 10 mg/mL BSA (Promega) and 5 units of GoTaqFlexi DNA polymerase mix (Promega). The 16S gene was amplified using the primer pair 16Sar/16Sbr (Palumbi et al. 1991) or the pair 16SFt_amp (GCRGTATIYTRACYGTGCTAAGG) and 16SRt_amp2 (CTGGCTTAAACCGRTYTGAACTC) (K. Macdonald, pers. comm.). PCR reaction mix and cycling conditions for both gene regions were as follows: 35 cycles of 30 sec at 95° C, 30 sec at 50° C, 45 sec at 72° C. PCR products were purified with ExoSAP-IT (Affymetrix). Sequencing was performed using the BigDye Terminator 3.1 Cycle Sequencing Kit (Applied Biosystems).

Amphipod tissue samples of material from NorAmph, usually consisting of two or three pleopods, were prepared for the NORBOL-consortium following the procedures of the Barcode of Life Database (BOLD) system (Ratnasingham and Hebert 2007). These samples were sequenced in both directions by the Canadian Centre of DNA Barcoding (CCDB) using the primer pair C_LepFolF/C_LepFolR for both PCR and Sanger sequencing of COI (Hernández-Triana et al. 2014, Ratnasingham and Hebert 2007). The CCDB standard PCR for invertebrates is initial denaturation at 94°C for 2 min, 5 cycles of 94°C for 30 sec, annealing at 45°C for 40 sec, and extension at 72°C for 1 min, 35 cycles of 94°C for 30 sec, annealing at 51°C for 40 sec, and extension at 72°C for 1 min, and a final extension at 72°C for 10 min. Voucher specimens for these sequences are stored in the Invertebrate Collections of the University Museum of Bergen, Norway. Sequences with voucher pictures and metadata are available from the BOLD website (boldsystems.org) using the accession codes provided in Table 1 and Suppl. material 1, or https://doi.org/10.5883/DS-RHACHOTR.

In addition to these new sequences, we used previously published sequences from *Rhachotropis* (Lörz et al. 2012) and *Eusirus holmii* as outgroup in the phylogenetic analyses.

Sequences were assembled with the software package Geneious (version 10.0.9) (Kearse et al. 2012) and aligned with the MAFFT plugin (Katoh and Standley 2013, Katoh et al. 2002).

We used MEGA7 (Kumar et al. 2016) to group sequences according to morphology based identifications and to compute p-distances within and between groups with standard errors estimated from 1000 bootstrap replicates.

We used FastTree2 ver. 2.1.5 (Price et al. 2010) with the GTR plus gamma model and 20 rate categories to estimate initial gene trees from the sequences. Support values for nodes were calculated with the Shimodaira-Hasegawa (Shimodaira and Hasegawa 1999) test and 1000 resamples.

We used MrBayes ver 3.2 (Huelsenbeck and Ronquist 2001, Ronquist and Huelsenbeck 2003) with the mixed gamma function command to simultaneously search for the optimal model and tree set by sampling across the space of the General Time Reversible model (GTR). The data were partitioned into two parts, one comprised by first and second codon positions, the second by third codon positions. Model parameters were estimated independently for the two. Two million generations for two parallel runs of four chains sampling every 1000 generations were monitored with the Average Standard Deviation of Split Frequencies (ASDSF) in MrBayes and with Tracer (Rambaut et al. 2013). Figtree (Rambaut 2014) was used for tree graphics.

Automatic Barcode Gap Discovery (ABGD) (Puillandre et al. 2012) was run via the web interphase at http://wwwabi.snv.jussieu.fr/public/abgd/abgdweb.html, selecting simple distances and relative gap widths ranging from 1 via 1.5 (default) to 3.0 with FastA file input of the sequence data.

Additional sequences of *R. aculeata* were downloaded from BIN AAB3310 in BOLD. We calculated a Median Joining Network with POPART (Leigh and Bryant 2015).

Geographic distances (in km) between the samples were calculated with Geographic Distance Matrix Generator (Ersts 2017).

## Results

### Systematics

**Order AMPHIPODA Latreille, 1816**

**Suborder GAMMARIDEA Latreille, 1802**

**Family EUSIRIDAE Stebbing, 1888**

**Genus *Rhachotropis* S.I. Smith, 1883**

***Rhachotropis* S.I. Smith, 1883: 222.**

***Gracilipes* Holmes, 1908: 526.**

**Figure 1.**
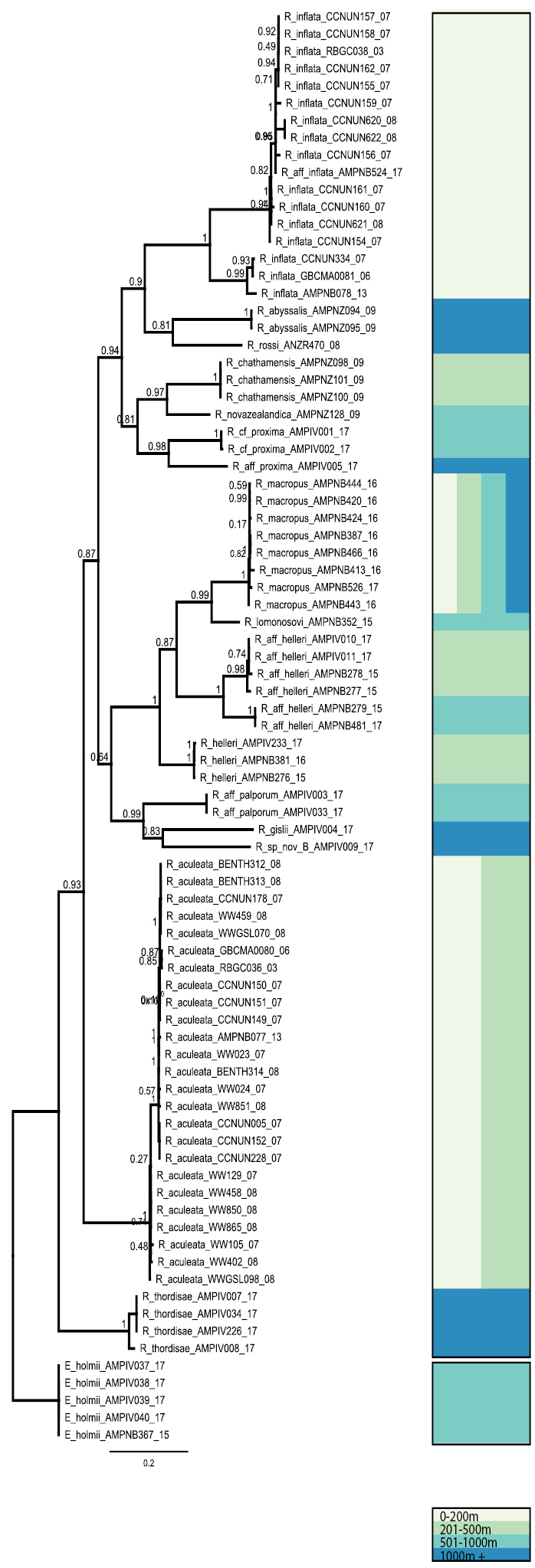
COI gene tree calculated with FastTree2 ver. 2.1.5 (Price et al. 2010) with the GTR plus gamma model and 20 rate categories of sites to estimate initial gene trees from the sequences. Support values were calculated for nodes with the Shimodaira-Hasegawa (Shimodaira and Hasegawa 1999) test and 1000 resamples. Samples via IceAGE, NorAmph and mined from Genbank (Details Supplementary Table 1). Clades are coloured for depth strata for sampling: 0–200 m light grey, 201–500 m light green, 501–1000 m turquoise, 1000+ m blue. A combination of vertical colours indicates a combination of depth strata.

We obtained sequences from 42 *Rhachotropis* specimens in our samples (Table 1). Thirty-one newly obtained sequences of COI were combined with 53 sequences available in GenBank (Fig. 1). All available information for the GenBank data, such as sampling details, can be found in the Suppl. material 1. Our new COI sequences were assigned to 13 different Barcode Index Numbers (BINs) in BOLD, of which all except AAB3310 (*Rhachotropis aculeata*) were new to the database. This greatly increased the representation of the diversity of this genus in the BOLD database.

**Table 1.** Overview of Rhachotropis sequences produced for this work, with BOLD accession numbers and BIN numbers (BOLD). The dataset can be accessed using https://doi.org/10.5883/DS-RHACHOTR

Species name	BOLD number	16S	COI	BIN number (BOLD)
*Rhachotropis aculeata* (Lepechin, 1780)	AMPIV200-17	x	x	AAB3310
*Rhachotropis aculeata* (Lepechin, 1780)	AMPNB077-13		x	
*Rhachotropis aff inflata* (Sars, 1883)	AMPNB524-17		x	–
*Rhachotropis aff palporum* Stebbing, 1908	AMPIV033-17	x	x	ADH1827
*Rhachotropis aff palporum* Stebbing, 1908	AMPIV003-17	x	x	
*Rhachotropis aff proxima* Chevreux, 1911	AMPIV005-17	x	x	ADH1828
*Rhachotropis cf proxima* Chevreux, 1911	AMPIV001-17	x	x	ADH1784
*Rhachotropis cf proxima* Chevreux, 1911	AMPIV002-17	x	x	
*Rhachotropis gislii* Thurston, 1980	AMPIV004-17	x	x	ADH0956
*Rhachotropis aff helleri* (Boeck, 1871)	AMPIV010-17	x	x	ADE3179
*Rhachotropis aff helleri* (Boeck, 1871)	AMPIV011-17	x	x	
*Rhachotropis aff helleri* (Boeck, 1871)	AMPNB277-15		x	
*Rhachotropis aff helleri* (Boeck, 1871)	AMPNB278-15		x	
*Rhachotropis aff helleri* (Boeck, 1871)	AMPNB279-15		x	ADE1120
*Rhachotropis aff helleri* (Boeck, 1871)	AMPNB481-17		x	
*Rhachotropis helleri* (Boeck, 1871)	AMPIV233-17	x	x	ADE4377
*Rhachotropis helleri* (Boeck, 1871)	AMPNB276-15		x	
*Rhachotropis helleri* (Boeck, 1871)	AMPNB381-16		x	
*Rhachotropis inflata* (Sars, 1883)	AMPIV070-17	x		–
*Rhachotropis inflata* (Sars, 1883)	AMPNB078-13		x	ACF8625
*Rhachotropis lomonosovi* Gurjanova, 1934	AMPNB352-15		x	ACW7325
*Rhachotropis macropus* Sars, 1893	AMPNB413-16		x	ADD5182
*Rhachotropis macropus* Sars, 1893	AMPNB420-16		x	
*Rhachotropis macropus* Sars, 1893	AMPNB424-16		x	
*Rhachotropis macropus* Sars, 1893	AMPNB387-16		x	
*Rhachotropis macropus* Sars, 1893	AMPNB443-16		x	
*Rhachotropis macropus* Sars, 1893	AMPNB444-16		x	
*Rhachotropis macropus* Sars, 1893	AMPNB466-16		x	
*Rhachotropis macropus* Sars, 1893	AMPNB526-17		x	
*Rhachotropis northriana* d’Udekem d’Acoz, Vader & Legezinska, 2007	AMPIV227-17	x		–
*Rhachotropis northriana* d’Udekem d’Acoz, Vader & Legezinska, 2007	AMPIV224-17	x		
*Rhachotropis northriana* d’Udekem d’Acoz, Vader & Legezinska, 2007	AMPIV225-17	x		
*Rhachotropis northriana* d’Udekem d’Acoz, Vader & Legezinska, 2007	AMPIV231-17	x		
*Rhachotropis northriana* d’Udekem d’Acoz, Vader & Legezinska, 2007	AMPIV230-17	x		
*Rhachotropis oculata* (Hansen, 1887)	AMPIV228-17	x		–
*Rhachotropis* sp. n. B	AMPIV009-17	x	x	ADH1829
*Rhachotropis thordisae* Thurston, 1980	AMPIV034-17	x	x	ADH0957
*Rhachotropis thordisae* Thurston, 1980	AMPIV007-17	x	x	
*Rhachotropis thordisae* Thurston, 1980	AMPIV008-17	x	x	
*Rhachotropis thordisae* Thurston, 1980	AMPIV226-17	x	x	
*Rhachotropis thorkelli* Thurston, 1980	AMPIV006-17	x		–
*Rhachotropis thorkelli* Thurston, 1980	AMPIV078-17	x		

**Figure 2.**
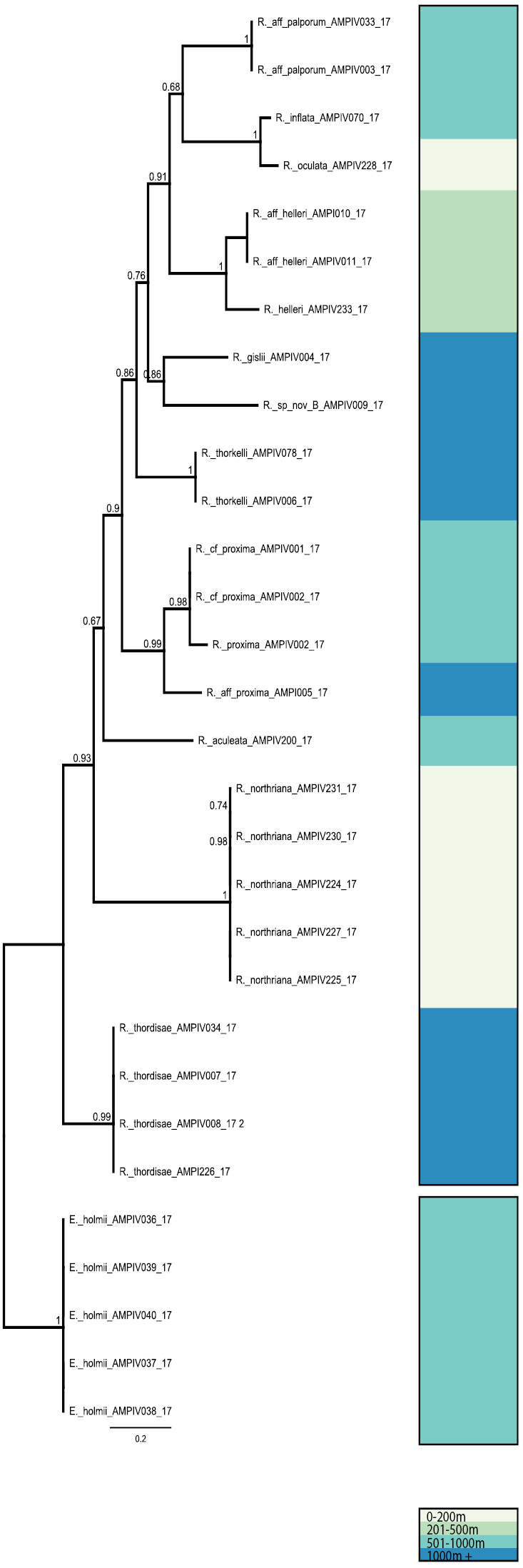
16S gene tree calculated as in Fig. 1- *Rhachotropis* samples collected during IceAGE (details Supplementary Table 1). Clades are coloured for depth strata for sampling: 0–200 m light grey, 201–500 m light green, 501–1000 m turquoise, 1000+ m blue.

Twenty-four *Rhachotropis* 16S sequences were generated from the recent IceAGE collections and analysed separately (Fig. 2).

Within and between-group mean p-distances with estimated standard errors are shown in Tables 2 and 3 respectively. Most of the groups are about 20 % different in pairwise comparisons. Exceptions are *R. macropus* and *R. lomonosovi* with a difference of only 9.8 % and *R. inflata* and R. aff. inflata with a 3.5 % difference. Within group mean distances show relatively homogenous groups: 8.7 % difference in *R. thordisae*, 5.8 % in *R. inflata*, and 5 % in R. aff. helleri. The BOLD BINs display results congruent with our morphological investigation: the two R. cf proxima sequences belong to a different BIN than the R. aff. proxima (Table 1) with a p-distance of 0.15 (Table 3). The R. aff helleri sequences were assigned to two different BINs by BOLD. The two groups were calculated with a p-distance of 8.56 % and came out as sisters in the COI gene trees (Fig. 1, Suppl. material 4), but not as monophyletic with the one we consider as the genuine *R. helleri*. The latter was assigned to a third BIN and is 15.7 % different from the former group (Table 3).

**Table 2.** Estimates of Average Evolutionary Divergence over Sequence Pairs within morphologically defined groups. The number of base differences per site from averaging over all sequence pairs within each group are shown. Standard error estimate(s) are shown in the last column. The analysis involved 82 nucleotide sequences. Codon positions included were 1st+2nd+3rd. All ambiguous positions were removed for each sequence pair. There were a total of 648 positions in the final dataset. Evolutionary analyses were conducted in MEGA7. The presence of n/c in the results denotes cases in which it was not possible to estimate evolutionary distances.

Species	p-dist	std_err
*Eusirus holmii*	0.000	0.000
*Rhachotropis abyssalis*	0.000	0.000
*Rhachotropis aculeata*	0.009	0.002
*Rhachotropis aff helleri*	0.050	0.005
*Rhachotropis aff inflata*	n/c	n/c
*Rhachotropis aff palporum*	0.000	0.000
*Rhachotropis aff proxima*	n/c	n/c
*Rhachotropis cf proxima*	0.002	0.001
*Rhachotropis chathamensis*	0.000	0.000
*Rhachotropis gislii*	n/c	n/c
*Rhachotropis helleri*	0.000	0.000
*Rhachotropis inflata*	0.058	0.005
*Rhachotropis lomonosovi*	n/c	n/c
*Rhachotropis macropus*	0.004	0.001
*Rhachotropis novazealandica*	n/c	n/c
*Rhachotropis rossi*	n/c	n/c
*Rhachotropis* sp n. B	n/c	n/c
*Rhachotropis thordisae*	0.011	0.003

ABGD analyses returned 18 groups of *Rhachotropis* when using default relative gap width of 1.5 (Suppl. material 3), all corresponding to the clusters found with the phylogenetic analyses. When increasing the gap width to three, *R. aculeata* was additionally divided into two groups (not shown).

Model testing of the data in two partitions using the Bayesian Information Criterion (BIC) resulted in the TN93+G model both for the third codon position and for the combination of the first and second. Similar models were obtained with MrBayes.

Phylogenetic estimates with MrBayes and FastTree based on COI sequences returned very similar tree topologies (Fig. 1, Suppl. material 4.) with few exceptions, such as the position of *R. helleri*. Some of the relationships between species had posterior probabilities lower than 0.90 and were therefore not well supported.

The FastTree estimate based on 16S data was indicating somewhat similar tendencies, such as the early divergence of *R. thordisae* and the splits in the groups associated with *R. proxima* and *R. helleri*. The two gene trees were otherwise difficult to compare because the 16S data set included sequences from *R. northriana*, *R. oculata*, *R. aculeata* and *R. thordisae*, for which COI are missing. Likewise, 16S sequences were not obtained for many of the taxa represented in the COI data set, including *R. macropus*, *R. lomonosovi*, *R. gislii* and others (Fig. 2).

**Figure 3.**
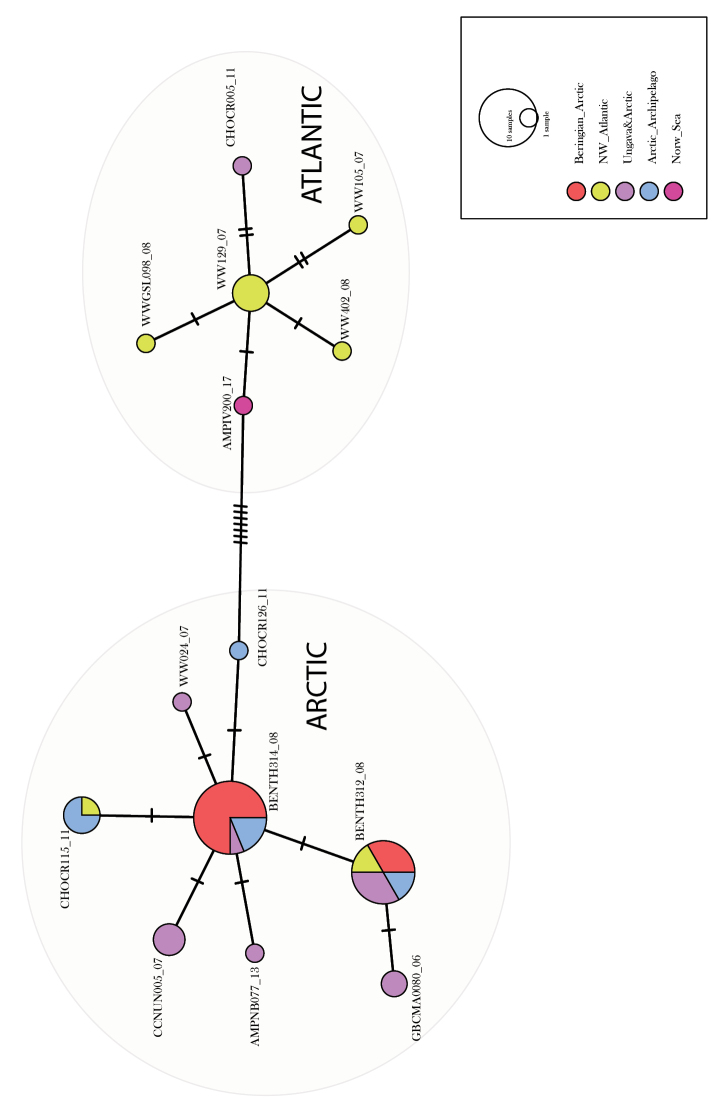
Median Joining haplotype network of *Rhachotropis aculeata* calculated with POPART (Leigh and Bryant 2015) using five geographical clusters.

Additional COI sequences of *R. aculeata* downloaded from BIN AAB3310 in BOLD show some geographic structure. We calculated a Median Joining Network with five geographical groups in POPART (Leigh and Bryant 2015). The species appears to have a special cluster in the Atlantic, including the Canadian West Atlantic and Norwegian Sea, whereas the Svalbard specimen clusters with the Arctic populations of North America (Fig. 3).

**Figure 4.**
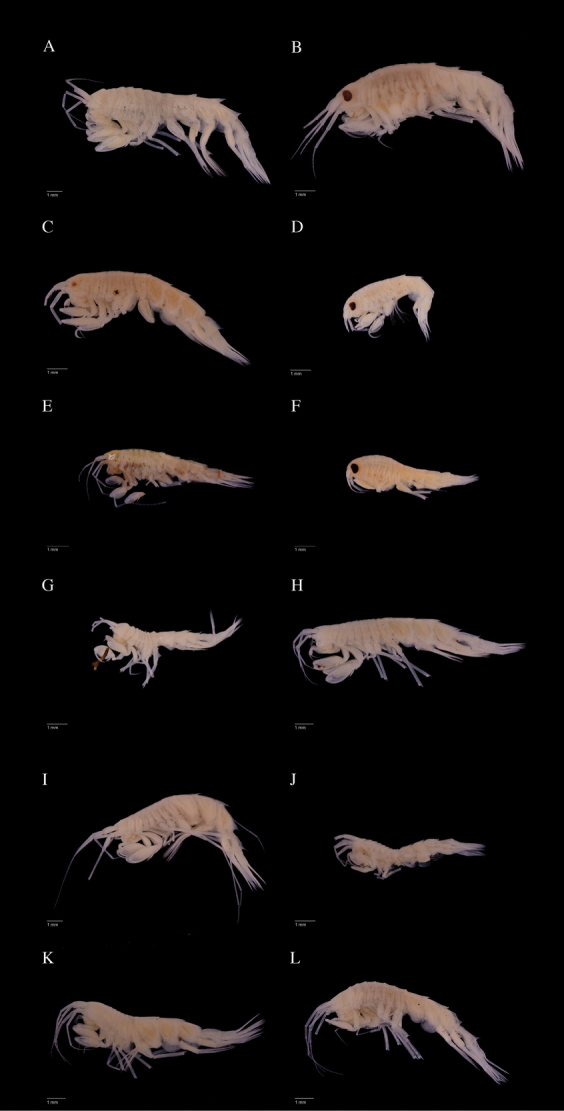
Lateral view photos of IceAGE material representing different clades in the analyses. **A** *Rhachotropis gislii* AMPIV004-17 **B** *R. helleri* AMPIV233-17 **C** R. aff. helleri AMPIV011-17 **D** *R. inflata* AMPIV070-17 **E** *R. northriana* AMPIV225-17 **F** *R. oculata* AMPIV228-17 **G** R. aff. palporum AMPIV033-17 **H** R. aff. proxima AMPIV005-17 **I** R. cf. proxima AMPIV002-17 **J** *R.* sp. B AMPIV009-17 **K** *R. thordisae* AMPIV007-17 **L** *R: thorkelli* AMPIV078-17.

**Figure 5.**
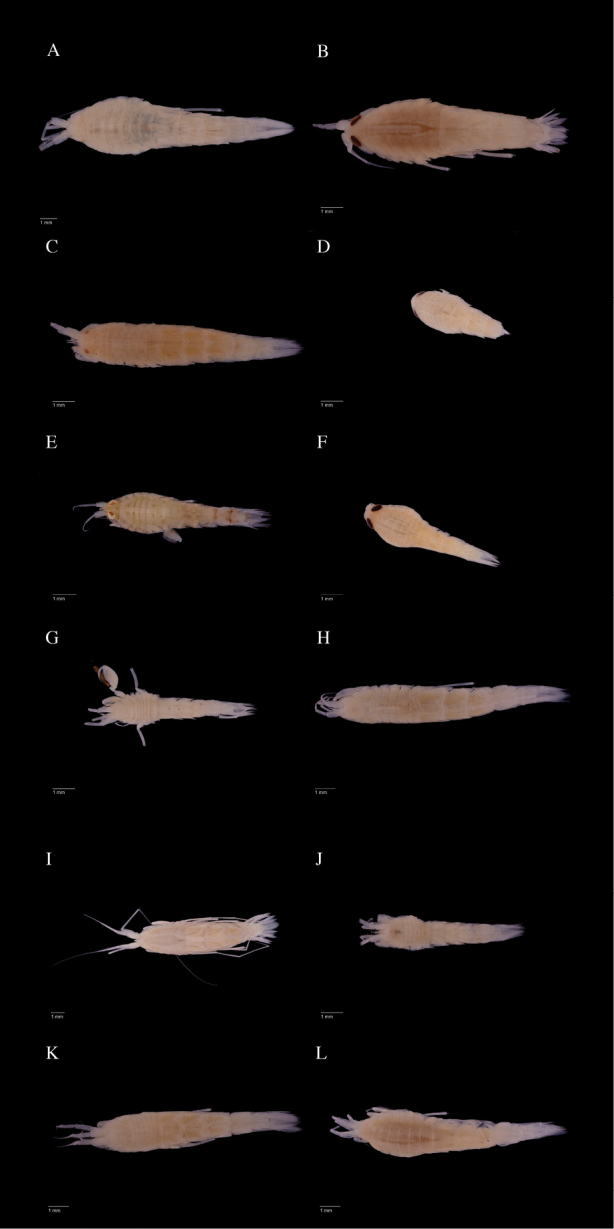
Dorsal view photos of IceAGE material representing different clades in the analyses. **A** *Rhachotropis gislii* AMPIV004-17 **B** *R. helleri* AMPIV233-17 **C** R. aff. helleri AMPIV011-17 **D** *R. inflata* AMPIV070-17 **E** *R. northriana* AMPIV225-17 **F** *R. oculata* AMPIV 228-17 **G** R. aff. palporum AMPIV033-17 **H** R. aff. proxima AMPIV005-17 **I** R. cf. proxima AMPIV002-17 **J** *R.* sp. B AMPIV009-17 **K** *R. thordisae* AMPIV007-17 **L** *R: thorkelli* AMPIV078-17.

**Figure 6.**
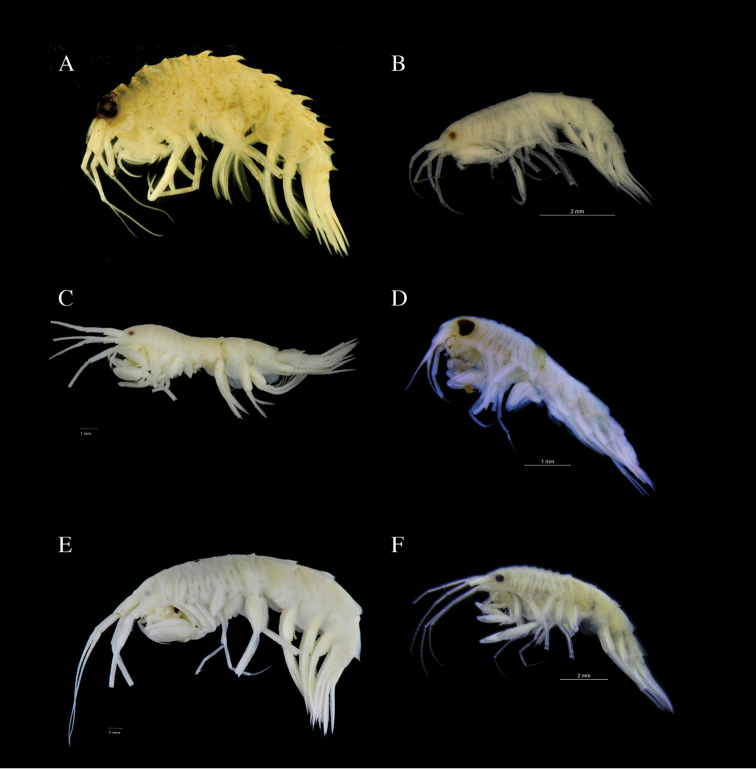
Lateral view photos of NorAmph material representing different clades in the analyses. **A** *Rhachotropis aculeata* AMPNB077-13 **B** *R. helleri* AMPNB276-15 **C** R. aff. helleri AMPNB279-15 **D** R. aff. inflata AMPNB524-17 **E** *R. lomonosovi* AMPNB352-15; F. *R. macropus* AMPNB443-16.

Photographs were taken of at least one representative of each clade (Figs 4–6).

## Discussion

Overall the morphological differentiation of the *Rhachotropis* sampled in the NE Atlantic is mirrored in the differentiation of our mitochondrial DNA markers and reflected both in gene tree topology and genetic distances. However, both R. aff. helleri and *R. inflata* have diverged into groups that were unnoticed *a priori* from morphology and R. aff. inflata clusters with one of the latter lineages. The taxonomic status of R. aff. helleri versus *R. helleri* must be examined further with more data. There is also considerable COI divergence in the *R. proxima* group.

Our DNA sequence data are shedding new light on the species relationships of *Rhachotropis*, although based on one gene fragment only the phylogenetic trees should certainly be interpreted with caution. The difference between gene trees and species trees has been an important topic in theoretical phylogenetics since the seminal publication by Maddison (1997). The phenomenon of lineage sorting and deep coalescence certainly also has implications for our understanding of species, particularly with respect to the idea of divergent monophyletic lineages and of species delimitation based on a single mitochondrial marker (Degnan and Rosenberg 2006, 2009, Knowles and Kubatko 2010). While the DNA barcoding campaign has sparked an excess of new species discoveries, it should be remembered that gene trees are representation of genetic structure and that non-monophyletic or genetically distant subclades are not necessarily separate species (Sukumaran and Knowles 2017). We have found that there is generally good correspondence between morphological species assignments and genetic structure in *Rhachotropis* but that there are also several examples of haplotype clusters that are candidates for broader systematic examination with multiple genes and morphological analyses.

The following discussion is divided according to three questions asked.

*1) What are the phylogenetic relationships of North East Atlantic and Arctic Rhachotropis*?

The FastTree approach and the Bayesian method returned very similar tree topologies with minimum exceptions. In both cases there was strong support for most of the species clades and also for some sister species relationships. However, many of the deeper branches were less well supported, which should be kept in mind when inferring the evolutionary history of ecological and biogeographical events.

Only three species occurred in both sampling sets from our two collecting groups in Icelandic and Norwegian waters: *R. aculeata* (Fig. 6A), *R. helleri* (Figs 4B, 5B, 6B) and *R. inflata* (Figs 4D, 5D). Eight species were only sampled during the IceAge project, four species only during NorAmph. However, we assume that the species collected in certain depth strata could be found in the corresponding depth area of other areas. We assume that depth and temperature have a greater effect than other characteristics, e.g. sediment type; for example *Rhachotropis helleri* was found on the Reykjanes ridge in 289 m (Fig. 4B), the Lofoten area off Norway in 850 m, in the Sognefjord 307 m (Fig. 6B) and the Norwegian channel 203 m (see Table 1). The dominant sediment of the Reykjanes ridge is muddy sand with a small proportion of gravel (Meißner et al. 2014), whereas the *R. helleri* off Norway were collected on sediments dominated by sandy mud with little gravel.

Weisshappel (2000) investigated the biogeographic distribution of Eusiridae species around Iceland and found a marked separation along the Greenland-Iceland-Faroe (GIF) Ridge. Weisshapel assumed that eusirids followed relatively sharp boundaries between water masses of different origin lying on and adjacent to the GIF ridge or within the area on each side of the ridge.

Weisshappel (2000) showed that most eusirid species are restricted to certain combinations of bottom water temperatures and bottom depths, 28 out of 36 were found within a single water mass. Different Eusiridae species show different biogeographic preferences. *Eusirus holmii*, for example shows a very narrow temperature distribution – it is only collected in waters colder than 1°C – but has a very wide depth range, 400 to 1600 m. *R. aculeata* is the opposite: it has a wide temperature tolerance (-1°C to +6°C), and a relative narrow depth distribution, 100-600 m. There seems to be some divergence (Fig. 3) between Atlantic and Arctic populations. Whereas the *R. aculeata* specimen from Spitzbergen Isfjord at 123 m cluster with the latter group, the *R. aculeata* from 580 m in the Norwegian Sea is closer to the COI haplotypes occurring in Canadian West Atlantic waters.

An even more temperature-tolerant species is *R. inflata* which only occurs in shallow waters above 400 m, but tolerating temperatures from 0°C to 9°C (Weisshappel 2000 and current study). Morphological separation of *R. inflata* (Figs 4D, 5D) and R. aff. inflata (Fig. 6D) is minute: a dent on urosomite 1 in contrast to a non-dented urosomite 1. When applying these two morphology based groups in computation of p-distance, the mean divergence is only 3.5 % (Table 3). However, COI data divides *R. inflata* into two distinct clusters, one also including R. aff. inflata, the latter which clusters with sequences of *R. inflata* from Nunavut. The mean p-distance between the two *R. inflata* clades is actually 15 %, so these clades are clearly candidates for critical taxonomic examination with multiple gene data. Not enough material was available for a study of the character development in different growth stages in both genders within the framework of this study.

**Table 3.** Estimates of evolutionary divergence (p-distance) over sequence pairs between groups. The number of base differences per site from averaging over all sequence pairs between groups are shown. Standard error estimate(s) are shown above the diagonal. The analysis involved 82 nucleotide sequences. Codon positions included were 1st+2nd+3rd. All ambiguous positions were removed for each sequence pair. There were a total of 648 positions in the final dataset. Evolutionary analyses were conducted in MEGA7.

*Eusirus holmii*		0.014	0.015	0.015	0.023	0.015	0.015	0.015	0.015	0.015	0.016	0.014	0.016	0.016	0.014	0.016	0.015	0.014
*Rhachotropis abyssalis*	0.260		0.014	0.014	0.023	0.014	0.014	0.013	0.014	0.016	0.016	0.013	0.015	0.015	0.014	0.014	0.014	0.015
*Rhachotropis aculeata*	0.211	0.217		0.013	0.022	0.014	0.014	0.014	0.014	0.014	0.015	0.013	0.013	0.014	0.014	0.015	0.015	0.013
*Rhachotropis aff helleri*	0.238	0.247	0.217		0.022	0.014	0.014	0.013	0.014	0.014	0.012	0.013	0.011	0.011	0.015	0.014	0.015	0.013
*Rhachotropis aff inflata*	0.236	0.198	0.207	0.242		0.022	0.022	0.022	0.021	0.021	0.029	0.005	0.023	0.024	0.021	0.022	0.023	0.022
*Rhachotropis aff palporum*	0.218	0.237	0.194	0.237	0.194		0.014	0.015	0.014	0.014	0.014	0.014	0.014	0.015	0.015	0.015	0.014	0.015
*Rhachotropis aff proxima*	0.233	0.187	0.194	0.222	0.220	0.224		0.012	0.013	0.015	0.015	0.013	0.014	0.013	0.013	0.015	0.014	0.014
*Rhachotropis cf proxima*	0.221	0.191	0.201	0.222	0.204	0.212	0.152		0.014	0.015	0.015	0.014	0.014	0.014	0.014	0.015	0.014	0.015
*Rhachotropis chathamensis*	0.204	0.219	0.194	0.219	0.164	0.195	0.185	0.187		0.015	0.016	0.013	0.014	0.015	0.012	0.016	0.015	0.014
*Rhachotropis gislii*	0.239	0.233	0.221	0.237	0.210	0.207	0.242	0.225	0.224		0.016	0.014	0.014	0.014	0.015	0.015	0.014	0.015
*Rhachotropis helleri*	0.215	0.245	0.197	0.157	0.242	0.192	0.228	0.213	0.215	0.228		0.015	0.013	0.015	0.015	0.015	0.016	0.015
*Rhachotropis inflata*	0.249	0.223	0.229	0.242	0.035	0.222	0.237	0.224	0.211	0.236	0.241		0.014	0.014	0.012	0.014	0.015	0.014
*Rhachotropis lomonosovi*	0.255	0.258	0.219	0.164	0.210	0.216	0.233	0.225	0.210	0.238	0.150	0.236		0.011	0.014	0.015	0.015	0.013
*Rhachotropis macropus*	0.246	0.247	0.219	0.158	0.222	0.223	0.230	0.238	0.226	0.249	0.163	0.250	0.098		0.013	0.015	0.015	0.014
*Rhachotropis novazealandica*	0.222	0.207	0.202	0.225	0.204	0.218	0.182	0.174	0.129	0.235	0.226	0.216	0.207	0.236		0.015	0.015	0.014
*Rhachotropis rossi*	0.235	0.188	0.213	0.234	0.211	0.242	0.223	0.197	0.220	0.242	0.251	0.231	0.237	0.218	0.203		0.016	0.015
*Rhachotropis* sp n. B	0.248	0.256	0.214	0.241	0.246	0.196	0.236	0.234	0.222	0.219	0.244	0.262	0.221	0.256	0.218	0.244		0.015
*Rhachotropis thordisae*	0.208	0.248	0.194	0.211	0.206	0.226	0.240	0.219	0.210	0.223	0.206	0.222	0.205	0.207	0.198	0.222	0.244	

DNA barcoding revealed three clades within a species that keyed out to *Rhachotropis helleri*. The original description of *R. helleri* was provided by Boeck (1871); one brief paragraph in latin with no illustrations. Sars (1895) illustrated *R. helleri* in detail. We regard the specimens illustrated and described by Sars as corresponding to the species named by Boeck. Further morphological investigation of our *R. helleri* samples revealed two *helleri* groups. The first group has a smooth dorsal seventh pereonite and eyes larger than 50 % of head (see Figs 4B, 5B, 6B) – this we named *R. helleri* as it closely matches the drawings provided by Sars (1895). The second group specimens bear a small distinct spine on their seventh pereonite and have a smaller eye, covering a maximum of 20 % of the head. We provisionally named these R. aff. helleri (Figs 4C, 5C, 6C). The specimens of the R. aff. helleri group split into two molecularly distinct clades (Fig. 1, Suppl. material 3) with a p-distance of 8.56 %. We were not able to find a morphological differentiation in this group.

We conclude that the two aff. helleri groups therefore either represent a single species, which contains widely-divergent COI sequence, or that the two clades may be two species, which are genuinely cryptic rather than pseudo-cryptic (Janosik and Halanych 2010; Brandt et al. 2014). Although there may be good reasons to flag potentially new species to attract further attention (Wägele 1994), we do not propose the formal erection of a new species here because the aff. helleri can be only distinguished by the presence-absence of one spine and eye size, characters that can vary with sex and body size, and the two aff. helleri can currently only be reliably identified by DNA sequencing.

*R. macropus* (Fig. 6F) and *R. lomonosovi* (Fig. 6E) show the smallest interspecific distance of 9.8 % (Table 2). *R. macropus* and *R. lomonosovi* are genetically (Table 3) and morphologically (Fig. 6E, 6F) very similar. *R. macropus* is known to be very numerous along the Norwegian coast and in Svalbard (d’Udekem et al. 2007). While generally found at moderate depths of 200-700 m, we have two specimens from 1230 m in our samples, which extends the depth range by 500 m. *R. lomonosovi* is a true Arctic species, common at depths of 1000-1500 m (d’Udekem et al. 2007). Our single representative of this *R. lomonosovi* is from the lower Arctic/ Norwegian northern coast (outer Lofoten area), from a depth of 823 m, but from a water temperature of -0.58°C.

*2) Do relationships between the shallow (Norwegian Channel) and the deep (Icelandic Basin and Norwegian Sea) amphipods indicate biogeographic processes such as submergence or emergence*?

Generally more species are currently known from the shelf and upper slope area. However, the observed depth pattern is heavily collection biased: areas with more stations show more species (Lörz et al. 2012). Bousfield and Hendrycks (1995) attempted a phylo-biogeographic analysis of *Rhachotropis* of the North Pacific. Some of the species, such as *R. aculeata*, *R. helleri*, *R. macropus*, *R. oculata*, *R. inflata* and *R. distincta* are known from both the North Pacific and the North Atlantic, but these distribution data are based on literature using morphological data only. Bousfield and Hendrycks (1995) postulated that species such as *R. aculeata* and *R. macropus* belong to more primitive forms. Characters considered plesiomorphic by Bousfield and Hendrycks (1995) were, for example, an elongate telson, long rostrum, strongly toothed pleon and urosome. These so-called primitive species have a large body size (10.5- 40 mm). In contrast the more advanced *oculata*-*inflata* groups have small body lengths (3.8-12 mm), smooth urosome and short telson. While the primitive large species were generally coastal shelf species, the species become smaller, more weakly spinose and anoculate in most abyssal members. Bousfield and Hendrycks (1995) assume submergence as the primary biogeographic pattern amongst the *Rhachotropis* species in general and amongst the North Pacific in particular. Our findings cannot confirm this. Neither can we agree that shallow = large, spiny species. Our most abundant species was collected at the shallowest station, *R. northriana*. This is a very small, not very spinose species with a relatively large telson. Several abyssal species are known to reach large body sizes, such as *R. rossi* (12 mm) or *R. abyssalis* (13 mm). Although some of the deeper nodes in our phylogeny (Fig. 1, Suppl. material 4) are not well supported, we also cannot agree that *R. aculeata* and *R. macropus* are the most primitive forms, as in our molecular analysis of COI and 16S they are not in a basal position. When observing depth distributions of *Rhachotropis* clades, there does not seem to a directional trend in terms of emergence or submergence of during speciation.

*Eyes*

**Figure 7.**
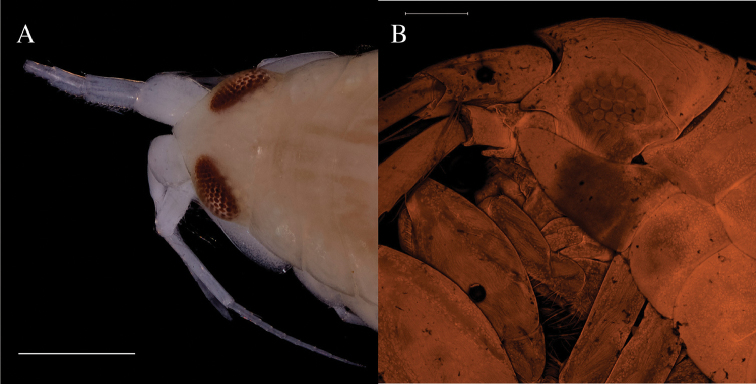
Eyes of, **A** *Rhachotropis helleri* AMPIV233-17, ommatidiae (Photo) **B** *Rhachotropis oculata* AMPIV228-17 (CLSM), 7 stack-layers have been removed in the eye-region to reveal the ommatidia beneath the cuticulum.

The genus *Rhachotropis* is known to have a diversity of “eye phenotypes”. *R. leucophthalma* G. O. Sars, 1893 is a white-eyed species – this feature is so prominent that it provided its name; its eyes become colourless and hard to see in alcohol (d’Udekem et al. 2007), whereas the true arctic species *R. lomonosovi* Gurjanova, 1934 also has white eyes when alive, but they turn black in alcohol. (This is sadly not easily seen in Fig. 6F, as the specimens cuticulum has loosened from the ommatidia leaving the eye colour an indistinct grey.) *Rhachotropis oculata* has kidney-shaped eyes which keep their colour, eg. AMPIV228-17 Figs 4F, 5F and for the ommatidia Fig. 7B. (Our specimens match the description of *R. oculata* by Hansen, 1887, but differ in various characters from *R. oculata* pictured by Bousfield and Hendrycks 1995.)

*Rhachotropis helleri* (AMPIV233-17) has distinct red eyes, even after being preserved in 98% ethanol for more than 4 years (Fig. 7A). The ommatidia are clearly hexagonal in shape. After 6 years of preservation the eye colour and the outline fade, e.g. AMPIV011-17 Figs 4C, 5C. Here there is a clear difference between the *R. helleri* (largish eyes, and clearly defined Figs 4B, 5B, 6B) and the R. aff. helleri (see also Fig. 6C) which have much smaller eyes and no clearly visible edge of the eye.

*R. northriana* has distinct red eyes also clearly visible after being preserved in ethanol for several years (eg. AMPIV225-17, Figs 4E, 5E).

Another species which maintains clearly visible dark eyes in preservation is *R. inflata* (eg. AMPIV070-17 Figs 4D, 5D).

We therefore think it unlikely that the ancestral *Rhachotropis* was a blind inhabitant of the deep sea. We assume that submergence has led to the loss of eyes in truly deep sea or abyssal species such as *R. thordisae* (Fig. 4K) and *R. thorkelli* (Fig. 4L). The latter species prove that *Rhachotropis* can hunt without eyesight, however all species collected in the photic zone have well developed eyes.

*3) Does depth have a bigger influence on the phylogeny of Rhachotropis than geographic distance*?

*Rhachotropis* specimens are found in all major oceans of the world: Arctic, Atlantic Ocean, Mediterranean Sea, Caribbean Sea, Indian Ocean, Pacific Ocean and the Southern Ocean (Lörz et al. 2012). *Rhachotropis* specimens have been collected in all water depths, from the shelf (e.g d’Udekem et al. 2007, Lowry and Springthorpe 2005, Lörz 2015) to abyssal and hadal sampling sites (Thurston 1980, Lörz 2010), in trenches (Dahl 1959), as well as around hydrothermal vents (e.g. Bellan-Santini 2006). Based on molecular data (COI) Lörz et al. (2012) predicted that depth had a greater influence on phylogeny than geographic distance.

The genetic distance between shallow species such as *Rhachotropis aculeata* from 600 m and deep sea species such as *R. thordisae* from 2750 m is only 16 %, the shallow water *R. inflata* from 123 m and the deep sea *R. thorkelli* is 20.7 %. The two deep sea species *R. gislii* and *R. thordisae* were collected at the same station at 2750 m depth and show a similar genetic distance of 22 % as the two species R. cf. proxima and R. aff. inflata (21%) both later collected around 900 m (Table 3). The smallest genetic distance was observed amongst the species *R. lomonosovi* and *R. macropus*, which were collected in 823 m and between 300 m and 1230 m respectively. Potentially our sampled depth band in the North Atlantic is not wide enough to sufficiently illuminate biogeographic questions. It is likely that samples from abyssal and hadal depths will heavily influence the interpretation of distribution patterns. However, when we include *Rhachotropis* samples from GenBank not collected in the NE Atlantic the lack of correlation between genetic distance and depth as well as the lack of correlation between genetic distance and geographic distance continues. *R. abyssalis* was sampled at 3210 m and its genetic distance to R. cf. proxima from South Iceland at 900 m is only 19 % – the same distance as to *R. rossi*, which was collected below 3000 m in the Ross Sea.

The intraspecific distance is around 5 % in R. aff. helleri, and less than 1 % in R. aff. palporum, *R. chathamensis* and *R. macropus*, even though the latter was sampled in a depth range of 330–1260 m. Recent investigations by Brix et al. (2014) of the eurybathic isopod species *Chelator insignis* off Iceland presented a different picture: the isopods collected in different depth strata had high genetic distances over 20%, indicating narrow vertical distribution ranges of cryptic species and highlighting bathymetry as key player in speciation processes. Jażdżewska et al. (2018) provide the first insight into cryptic amphipods off Iceland revealed by DNA barcoding.

A characteristic species, *R. aculeata* (Fig. 6A), was selected for further investigation via COI sequences available online. For a better visualization of genetic distances a haplotype network was calculated from the COI alignment (Fig. 3). The network shows 13 haplotypes of *R. aculeata*, but the maximum distance is only seven mutation steps between haplotypes, proving *R. aculeata* to have a remarkable truly circum-arctic distribution. The haplotype network shows that the Svalbard specimen clusters with the population in the Canadian Arctic (Fig. 3). The geographic distance between these population is thousands of km. Even though *Rhachotropis* are known to be good swimmers, this is a tremendous distribution for an animal with no larval stage. Due to their predatory feeding behaviour it is unlikely that *R. aculeata* clings to drifting algae or ice.

## Conclusion

Morphologically separated groups of *Rhachotropis* are well supported by the genetic markers COI and 16S, with possible cryptic species in Rhachotropis aff. helleri. We recommend a morphological study of allometry in this genus, where many species often are collected in large numbers. Our present data lead us to support the theory that *Rhachotropis* originated in shallow (photic) seas, and has subsequently submerged to greater (subphotic) depths, with loss of eyes for the abyssal species. The question about geographic versus bathymetric distance as a driver for genetic distance is harder to answer, as there is no clear picture for the entire genus.

## Supplementary materials

Supplementary material 1 Table S1. Extended overview of all samples used in analyses with geographical and environmental details Data type: Microsoft Excel Worksheet (.xlsx) Explanation note: Depths indicated in red have been found using the latitude and longitude information with the datapoint and the bathymetry-layer on Google Earth Pro. Depths indicated in blue are inferred from the general depths in the named (no latitude or longitude given) geographical location, given bathymetry-layer on Google Earth Pro. This is an open access article distributed under the terms of the Creative Commons Attribution License (CC BY 4.0), which permits unrestricted use, distribution, and reproduction in any medium, provided the original author and source are credited.Anne-Nina Lörz, Anne Helene Tandberg, Endre Willassen, Amy Driskell

Supplementary material 2 Table S2. Geographic distances between stations (sample name) in km. Data type: Microsoft Excel Worksheet (.xlsx) This is an open access article distributed under the terms of the Creative Commons Attribution License (CC BY 4.0), which permits unrestricted use, distribution, and reproduction in any medium, provided the original author and source are credited.Anne-Nina Lörz, Anne Helene Tandberg, Endre Willassen, Amy Driskell

Supplementary material 3 Figure S1. Tree showing COI sequence cluster derived from ABGD (Automatic Barcode Gap Discovery) analysis using simple distances and default parameter settings. Data type: Adobe Acrobat Document (.pdf) This is an open access article distributed under the terms of the Creative Commons Attribution License (CC BY 4.0), which permits unrestricted use, distribution, and reproduction in any medium, provided the original author and source are credited.Anne-Nina Lörz, Anne Helene Tandberg, Endre Willassen, Amy Driskell

Supplementary material 4 Figure S2. Bayesian consensus tree from COI sequences. Numbers on branches are posterior probabilities. See text for further information. Data type: Adobe Acrobat Document (.pdf) This is an open access article distributed under the terms of the Creative Commons Attribution License (CC BY 4.0), which permits unrestricted use, distribution, and reproduction in any medium, provided the original author and source are credited.Anne-Nina Lörz, Anne Helene Tandberg, Endre Willassen, Amy Driskell
