# Supplementary figures and images for: Rhachotropis (Eusiroidea, Amphipoda) from the North East Atlantic
Source: Zookeys. 2018 Jan 23;(731):75–101. doi: 10.3897/zookeys.731.19814 (PMC5810106; doi:10.3897/zookeys.731.19814)

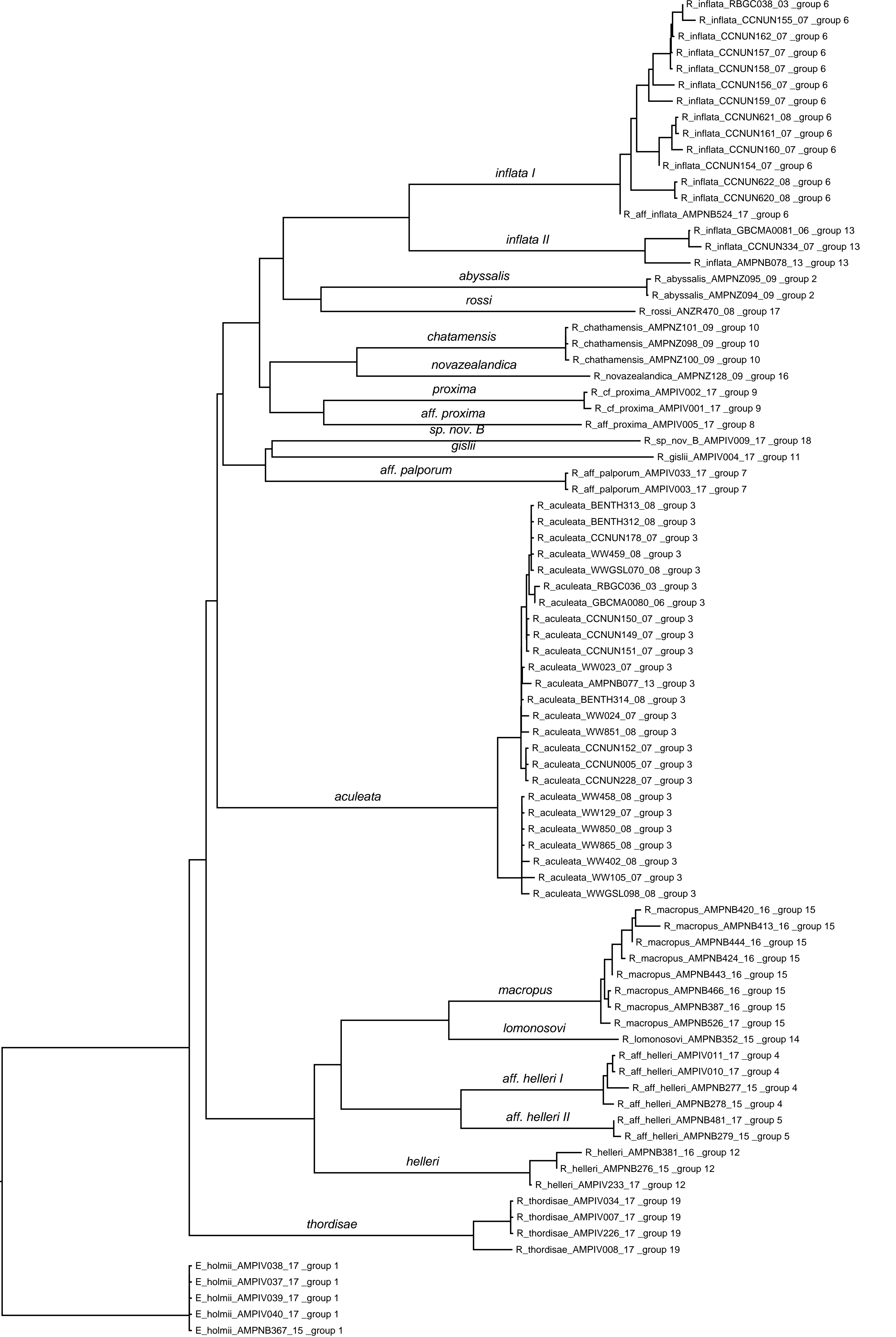

Supplement: Supplementary material 3 — Figure S1. Tree showing COI sequence cluster derived from ABGD (Automatic Barcode Gap Discovery) analysis using simple distances and default parameter settings. [file zookeys-731-075-s003.pdf]

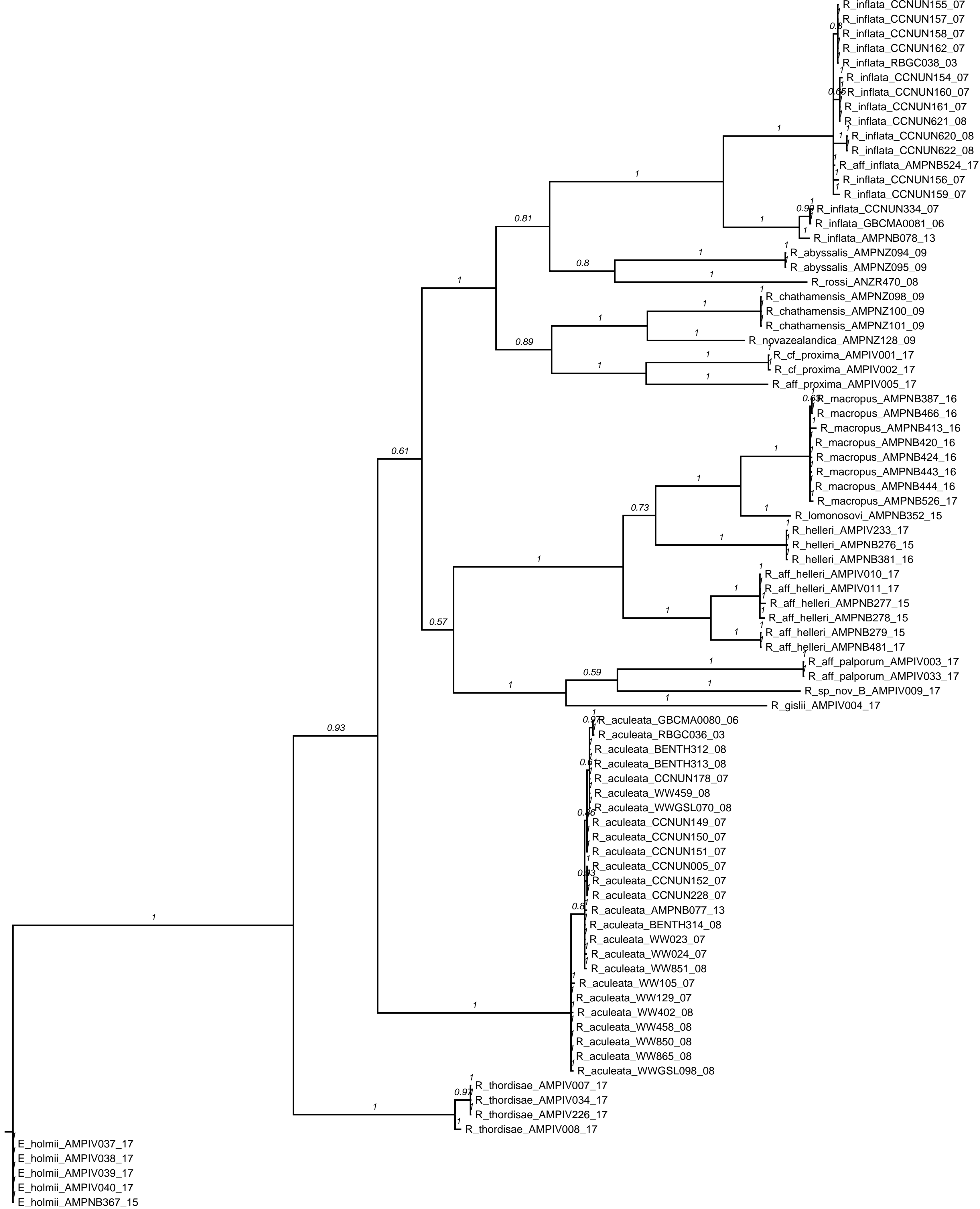

Supplement: Supplementary material 4 — Figure S2. Bayesian consensus tree from COI sequences. Numbers on branches are posterior probabilities. See text for further information. [file zookeys-731-075-s004.pdf]
